# Resting-State Functional Connectivity Predicts Attention Problems in Children: Evidence from the ABCD Study

**DOI:** 10.3390/neurosci5040033

**Published:** 2024-10-12

**Authors:** Kelly A. Duffy, Nathaniel E. Helwig

**Affiliations:** 1 Department of Psychology, University of Minnesota, 75 E River Road, Minneapolis, MN 55455, USA; 2 School of Statistics, University of Minnesota, 224 Church Street SE, Minneapolis, MN 55455, USA

**Keywords:** adolescent brain cognitive development (ABCD), attention-deficit/hyperactivity disorder (ADHD), functional magnetic resonance imaging (fMRI), group elastic net, Poisson regression

## Abstract

Attention deficit/hyperactivity disorder (ADHD) is a common neurodevelopmental disorder, and numerous functional and structural differences have been identified in the brains of individuals with ADHD compared to controls. This study uses data from the baseline sample of the large, epidemiologically informed Adolescent Brain Cognitive Development Study of children aged 9–10 years old (*N* = 7979). Cross-validated Poisson elastic net regression models were used to predict a dimensional measure of ADHD symptomatology from within- and between-network resting-state correlations and several known risk factors, such as biological sex, socioeconomic status, and parental history of problematic alcohol and drug use. We found parental history of drug use and biological sex to be the most important predictors of attention problems. The connection between the default mode network and the dorsal attention network was the only brain network identified as important for predicting attention problems. Specifically, we found that reduced magnitudes of the anticorrelation between the default mode and dorsal attention networks relate to increased attention problems in children. Our findings complement and extend recent studies that have connected individual differences in structural and task-based fMRI to ADHD symptomatology and individual differences in resting-state fMRI to ADHD diagnoses.

## 1. Introduction

### 1.1. ADHD Background

Attention-deficit hyperactivity disorder (ADHD) is a common neurodevelopmental disorder, affecting between 5 and 10% of school-aged children [1,2,3]. Neuroimaging studies have identified a number of differences at the group level in the brains of individuals with ADHD compared to controls, both structurally [4] and functionally. For studies using functional magnetic resonance imaging (fMRI), differences in particular brain regions have been identified using both task-based designs [5,6] and resting-state fMRI (rs-fMRI; [7]). However, in recent years, an increasing number of studies have focused on identifying entire brain networks that display altered activity, rather than individual regions. In rs-fMRI, this has been done primarily through functional connectivity studies, which evaluate correlated activity among different regions of the brain. Numerous differences in the network organization of individuals with ADHD have been identified, including reduced anticorrelations between task-related networks and the default mode network [8,9], reduced connectivity within the default mode network [10], and increased segregation and decreased integration between subnetworks across the whole brain [11,12].

In addition to brain differences, several other biological and social factors have been shown to be risk factors associated with ADHD. There is a strong genetic component of ADHD, which has an estimated heritability as high as 88% [13], so having a family member with ADHD increases one’s chances of having ADHD. Sex at birth is another important risk factor, such that males have higher rates of ADHD [2]. Another important risk factor for ADHD is socioeconomic status, such that those of lower socioeconomic status are more likely to have ADHD [14]. There also appears to be an association between parental substance use/abuse and ADHD, given that children of parents with substance abuse problems have been found to be more likely to be diagnosed with ADHD [15,16], and increased substance use has been reported in the parents of children in ADHD [17,18]. Although a number of neurobehavioral factors have been identified in both ADHD and substance use, such as increased reward sensitivity [19,20,21], deficits in executive function [22], and atypical default mode network activity [23,24,25], the exact underpinnings of this shared liability remain unclear.

### 1.2. Neuroimaging and ADHD

Despite the large number of neuroimaging findings related to ADHD and the identification of numerous risk factors, previous attempts to predict ADHD symptomatology and diagnoses using neuroimaging information have had only moderate success. Early success in small studies (e.g., [26]) has generally not been replicated as sample size has increased; in fact, sample size and accuracy are negatively correlated [27]. The ADHD-200 contest [28], presenting a huge progression in sample size by pooling data from multiple sites, challenged participating teams to create a diagnostic algorithm to classify ADHD (vs. controls) using imaging and demographic information. The average classification accuracy was 56.02% [29], and the best-performing algorithm obtained an accuracy of 61.54% [30]. Notably, however, the highest prediction accuracy was actually obtained by a team using only demographic information (e.g., gender, age, etc.; [31]). Predictive accuracies notably higher than chance have also been reported from the ADHD-ENIGMA study, a large consortium combining structural imaging information, but these authors used neural network models, which produce “black box” predictions that are challenging to interpret [32].

Classification studies based on known ADHD diagnoses, typically with clinically recruited samples, are common, but results from recent meta- and mega-analyses of imaging studies have started to call into question reliance on the case-control study paradigm. Mega-analyses of the ENIGMA study have found some reliable differences in brain volume and structure, but effect sizes are small (Cohen’s 0.11 <*d*< 0.21), and most differences are specific to children, and not found in adolescents and adults [4,33]. Several recent meta-analyses combining findings from numerous case-control studies in rs-fMRI have had conflicting results. Some spatial convergence of brain regions showing hyper- or hypo-connectivity was found when restricting analyses only to areas in networks of interest identified a priori [34,35], but a fully data-driven approach found no areas with significant spatial convergence [36]. The lack of consistent functional imaging findings generally [37] raises the question of whether the case-control approach may obscure significant heterogeneity that may exist within the brains of individuals with ADHD [36]. Approaches that focus more on individual differences, without requiring a binary classification into only two groups, may be more fruitful and generalizable.

Recently, Owens et al. [38] used data from the Adolescent Brain Cognitive Development (ABCD) study to predict a dimensional measure of ADHD symptomatology from structural MRI and task-based fMRI features. Using cross-validated elastic net regression [39], these authors found that structural MRI data explained about 0.7% of the variation in the ADHD symptomatology, whereas the task-based fMRI explained anywhere from 0% to 0.8% of the variation (depending on the task). After controlling for potentially confounding covariates, Owens et al. [38] found that the structural MRI had little predictive utility, whereas the functional fMRI retained some (reduced) predictive utility for certain tasks. Benefits of their approach include the use of a dimensional measure of ADHD (instead of a binary classification), as well as the use of a cross-validation-oriented regression methodology (instead of maximum likelihood). Limitations of their approach include the use of an atypical residualization and model selection procedure, which confounded the interpretation of the results. Furthermore, these authors only considered structural MRI and task-based fMRI, i.e., rs-fMRI connectivity was not considered.

### 1.3. Current Work

**Figure 1:**
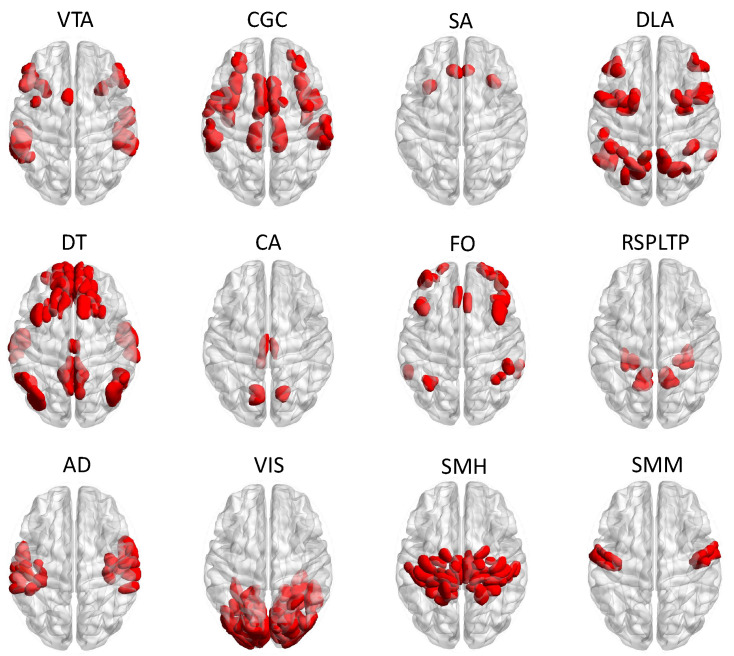
The 12 functional brain networks considered in this study. The brain networks were defined using regions of interest (ROIs) from the Gordon parcellation [42] and were plotted using the BrainNet Viewer software, version 1.7 [43]. The brain network abbreviations used in the ABCD Study^®^ include AD = auditory network, CA = cinguo-parietal network, CGC = cinguo-opercular network, DLA = dorsal attention network, DT = default network, FO = frontoparietal network, RSPLTP = restrosplenial temporal network, SA = salience network, SMH = somatomotor hand network, SMM = somatomotor mouth network, VIS = visual network, and VTA = ventral attention network.

**Figure 2:**
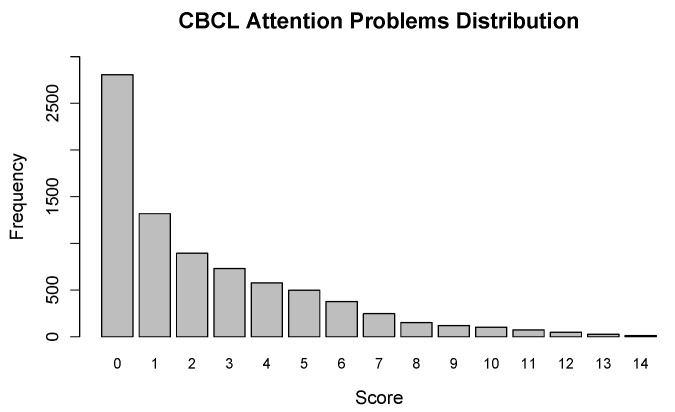
Histogram of the Attention Problems subscale of the Child Behavior Checklist (CBCL).

The aim of the present study is to explore the extent to which resting-state functional connectivity (rs-FC) is predictive of ADHD symptomatology. To accomplish this, we leveraged recent advances in elastic net regression [40] to predict ADHD symptomatology from within- and between-network resting-state correlations using data from the ABCD study. More specifically, we considered all pairwise combinations (including self-pairings) of the 12 networks in Figure 1, which produced 78 rs-FC features used in the group elastic regression analysis. As an outcome variable, we used the Attention Problems subscale of the Child Behavior Checklist (CBCL). Our goal was to predict these subscale scores from the 78 rs-FC features after controlling for common risk factors. It should be noted that the Attention Problems scores are non-negative integers with a right-skewed distribution (see Figure 2), so we used a Poisson response distribution. Based on recent work [41], we expected the rs-FC between the default mode network (DT) and the dorsal attention (DLA) to be most predictive of individual differences in ADHD symptomatology, as measured by the Attention Problems subscale of the CBCL, after controlling for common ADHD risk factors.

## 2. Materials and Methods

### 2.1. Participants

Subjects were participants in the ongoing ABCD study (www.ABCDstudy.org; accessed 30 August 2024), which collects data at 21 sites across the United States. Each data collection site obtained institutional review board (IRB) approval from their institutions, and study sites collected informed consent from parents and informed assent from subjects. Data from the baseline sample were used, meaning that participants ranged from 9 to 11 years of age (*M* = 9.940, SD = 0.628). In total, there were 11,878 participants in the baseline sample. Subjects were excluded for any type of relevant missing data, for rs-fMRI quality control (QC) failure, described in greater detail below, and for excessive motion during the rs-fMRI session, also clarified below. Following exclusion, there were *N* = 7979 subjects remaining, which were used in all following analyses. For demographic details of the sample, see Table 1.

### 2.2. fMRI Acquisition and Data Preprocessing

Anatomical and functional MRI scans were collected across the ABCD sites in accordance with the study protocol; for a thorough description of the acquisition parameters and the scanning protocol, please see Casey et al. [44]. Following collection, the rs-fMRI data were processed and QC was performed by the ABCD Data Analysis and Informatics Center (DAIC) in accordance with study protocol; for a thorough description of the processing and QC procedures, please see Hagler et al. [45]. See Appendix A for a brief treatment of fMRI data processing and QC procedures.

Regions of interest (ROIs) from the Gordon parcellation [42] were classified into 12 pre-determined functionally defined networks (plus a “none” category, consisting of ROIs that do not fit cleanly into any of the other networks, which was not included in analyses), which were cingulo-opercular (CGC), salience (SA), dorsal attention (DLA), ventral attention (VTA), default (DT), cingulo-parietal (CA), fronto-parietal (FO), retrosplenial temporal (RSPLTP), auditory (AD), visual (VS), somatomotor hand (SMH), and somatomotor mouth (SMM) networks; see Figure 1. It should be noted that network acronyms were chosen to be consistent with ABCD documentation, although some differ from the acronyms more standardly used throughout the literature. For ease of reference for these and the other acronyms used throughout the article, see the Abbreviations section (directly before Appendix A).

Within-network correlations were calculated as the average of the Fisher-transformed correlations across the time series for each unique, pairwise combination of ROIs belonging to the network. Average correlations between networks were calculated by averaging the correlations for each unique, pairwise combination of ROIs in the first network with the ROIs in the second. This process led to 78 total unique between- and within-network pairs across the 12 networks. It should be noted that time points with >0.2 mm framewise displacement or time periods with <5 contiguous frames below this framewise displacement threshold were not included in the calculation of correlations. We confirmed that all included subjects had at least 350 frames of data remaining after this censoring. Again, for a more detailed treatment of the calculation of the resting-state network correlations in ABCD, please see Hagler et al. [45].

### 2.3. Measures

Our outcome variable of interest, ADHD symptom scores, was determined as the sum score on the Attention Problems subscale of the CBCL, which evaluates multiple facets of ADHD symptomatology, including attention, hyperactivity, and impulsivity. Scores were the sum of the ratings on each item on the scale, with totals ranging from 0 to 14 (see Figure 2). This CBCL subscale has been demonstrated to be a good predictor of dichotomous ADHD diagnoses based on structured diagnostic interviews [46], and has been shown to have relatively high specificity and sensitivity in regards to clinical diagnoses [47]. However, although the scale shows a strong relationship to dichotomous diagnoses, scores were kept on a continuum to better assess ADHD along a spectrum of symptomatology, including sub-diagnostic individuals, to better evaluate individual differences. Due to the interest in more directly exploring ADHD along a continuum, this variable was selected as the outcome variable of interest over other categorical diagnostic variables collected as a part of the ABCD Study.

A demographic questionnaire was administered to the parent/caretaker of the participant to determine a number of demographic variables, including household combined income (coded as <50 k, 50–100 k, and >100 k, consistent with prior work in ABCD; [48]); highest parental education (coded as HS diploma or less, some college, Bachelor’s degree, and graduate degree, and taken to be the highest of either the responding parent or a partner who helps raise the child, if applicable); the child’s sex at birth (coded as either male or female, with three intersex males coded as male); the race the parent perceives the child to be (coded as White, Black/African-American, Native American/American Indian/Alaska Native, Asian/Asian-American/Pacific Islander, Hispanic/Latino [regardless of race identified], and multiple races/Other, where “Other” was a category selected by the parent). The participant’s age was determined based on the date they completed the rs-fMRI scan and was coded continuously. The study site of each participant was also included as a categorical predictor with 22 levels (21 active ABCD study sites plus one discontinued site).

A questionnaire was also administered to the parent/caretaker of the participant to assess the history of problematic drug (non-specified) or alcohol use in both biological parents as a lifetime occurrence. Problematic usage was defined as resulting in arrests or DUIs, harming health, necessitating treatment, and a number of other negative outcomes. Both variables were coded as either neither parent being affected, only the father being affected, only the mother being affected, or both parents being affected.

### 2.4. Data Analysis

To determine which demographic and/or brain connectivity features were predictive of the CBCL Attention problem scores, we used a group-penalized Poisson regression model [49]. We used the default (log) link function, so wemodeled the log of the expected CBCL score as an additive function of the predictors. For predictor variables, we used the previously described demographic measures (site, age, sex, race, income, and education), the parental substance use history variables (alcohol and drugs), and the 78 brain rs-FC measures. Five predictors were treated as unordered factors (i.e., site, sex, race, alcohol, drugs), two predictors were treated as ordered factors (i.e., income and education), and the remaining predictors were treated as numeric/continuous variables (i.e., age and 78 rs-FC measures).

The Poisson regression models were fit/tuned using the cv.grpnet() function in the **grpnet** R package [50], which implements the adaptively bounded gradient descent algorithm recently developed by [40]. In penalized, or “regularized,” regression, a penalty term is added to the likelihood function that penalizes the size of the estimated coefficients. The overall aim of penalization is to add a small amount of bias to the estimates such that the variance is reduced, due to the bias-variance trade off, and shrink the coefficient estimates closer to zero. This has the benefit of reducing model overfitting, and, if an $L_{1}$ penalty (based on an $L_{1}$ norm) is used, variable selection is also performed such that some coefficients are pushed completely to zero.

Unlike typical applications of penalized Poisson regression fit via the glmnet package [51], the grpnet package uses (i) a nonparametric regression approach that allows for nonlinear effects, and (ii) elastic net penalties that mix robust combinations of $L_{1}$ and $L_{2}$ penalties. More specifically, we expanded each feature using a spectral smoothing spline basis to capture nonlinear effects [49,52], and we compared three $L_{1}$ penalties: the least absolute shrinkage and selection operator (LASSO [53]), the smoothly clipped absolute deviation (SCAD [54]), and the minimax concave penalty (MCP [55]). The LASSO is the most commonly used $L_{1}$ penalty, but it has some noteworthy downsides, e.g., non-ignorable bias in the parameter estimates of the non-zero coefficients, particularly for larger effects [56,57], and a large number of false positive effects [57]. The SCAD and MCP penalties have been shown to have less bias and better selection properties and, thus, we elected to compare results using the three different penalty types.

The cv.grpnet() function uses 10-fold cross-validation to tune the model hyperparameters, which include the overall regularization parameter λ>0, the elastic net tuning parameter α∈[0,1], and the tuning parameter γ>2 (MCP) or γ>3 (SCAD). We used the defaults of the cv.grpnet() function, which tunes via a grid search using all combinations of α∈{0.01,0.25,0.5,0.75,1} and γ∈{3,4,5} (for MCP and SCAD) with the λ sequence automatically determined. We also used the default tuning measure, which is the mean absolute error (MAE) between the predicted and observed data. The combination of the hyperparameters that minimized the average MAE across the 10 folds was chosen.

## 3. Results

### 3.1. Cross-Validation

**Figure 3:**
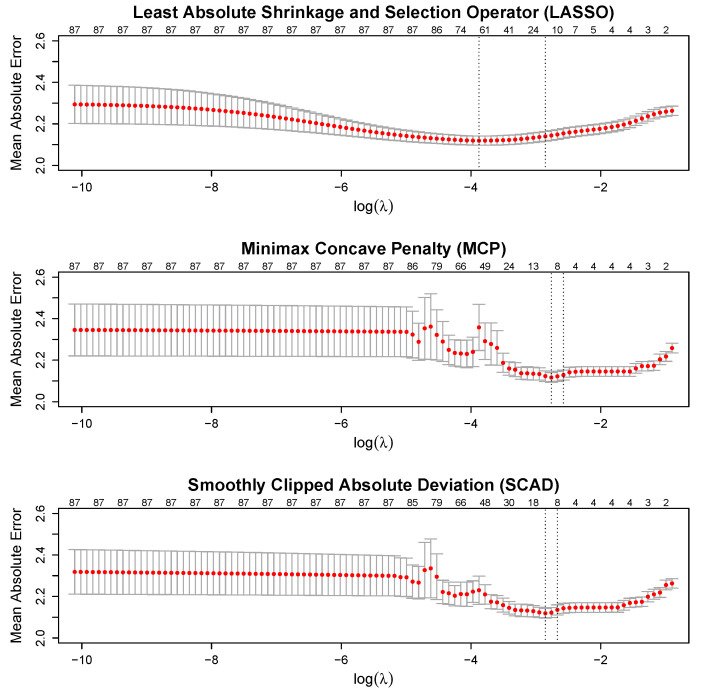
Prediction error curves for the three examined penalty functions. The *x*-axis represents the logarithm of each of the 100 λ values tested in cross-validation as part of the solution path. Red points show the average prediction error (operationalized as mean absolute error, and averaged across the 10 folds), whereas the gray lines display +/− one standard error. The two vertical (dotted) lines denote the solutions that minimize the prediction error (left) and the solution that is within one standard error of the minimum (right).

The cross-validation curves for the optimal hyperparameters (i.e., α and γ) are displayed in Figure 3. It should be noted that the red points show the average prediction error (averaged across the 10 folds), whereas the gray lines display +/− one standard error. The two vertical (dotted) lines denote the solutions that minimize the prediction error (left) and the solution that is within one standard error of the minimum (right). The “1se” solution is often preferred over the “min” solution, given that the 1se solution is more parsimonious and produces similar predictive performance as the min solution (e.g., see [51,58,59]).

### 3.2. Variable Selection and Importance

All three of the methods selected the following demographic/parental history variables: site, sex, income, alcohol, and drugs. The age and race variables were not selected by any of the methods, whereas the education variable was selected by the LASSO and the SCAD models (but not the MCP). Of the 78 rs-FC features included in the model, the MCP and SCAD only selected a single feature: the connection between the DT (default mode) and DLA (dorsal attention) networks. In contrast, the LASSO penalty selected the DT-DLA connection, as well as eight additional rs-FC network connections. However, it is important to note that a variable being selected by the model does not necessarily imply that the variable has a noteworthy influence on the solution.

**Figure 4:**
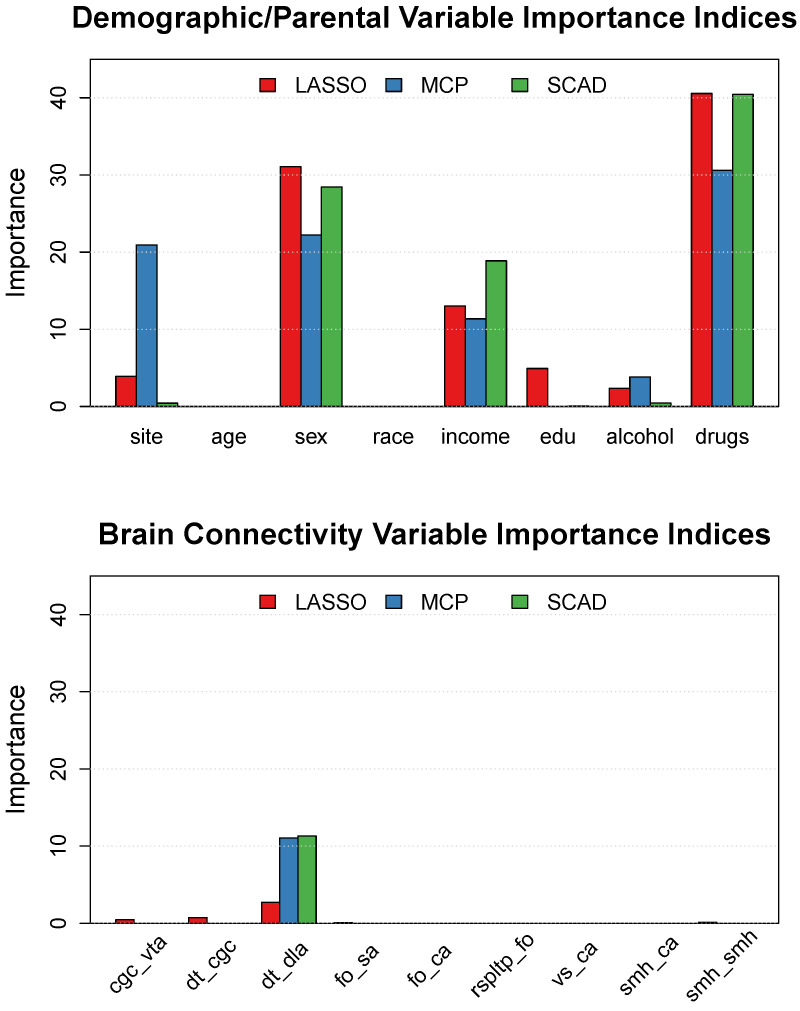
Variable importance indices for the demographic and parental history variables (**top**) and the active brain connectivity variables (**bottom**).

In nonparametric regression, the importance of each active (i.e., selected) term can be quantified via each term’s contribution to the variation accounted for by the model predictions (e.g., see [60]). Using the optimally tuned hyperparameters, the fit models explain the following proportions of the data variation (i.e., Poisson deviance): $R_{\mathrm{LASSO}}^{2}=8.516$%, $R_{\mathrm{MCP}}^{2}=9.430$%, and $R_{\mathrm{SCAD}}^{2}=7.928$%. The variable importance indices, which are plotted in Figure 4, give the approximate percentage of the overall $R^{2}$ that is accounted for by each term, so these values can be used to understand which terms are relatively more important for forming model predictions. It should be noted that the variable importance indices sum to 100 (across all variables separately for each penalty), so an importance value closer to 100 indicates that a given term is more important relative to the other terms in a given model.

All three models agree that parental history of problematic drug use (i.e., “drugs”) is the most important predictor of CBCL Attention Problems, with variable importance indices of LASSO = 40.564, MCP = 30.601, and SCAD = 40.436. After “drugs”, the biological “sex” of the child is the second most important predictor in all models: LASSO = 31.093, MCP = 22.226, and SCAD = 28.437. Interestingly, the MCP penalty attributes (a) less importance to the “sex” variable than the other penalties, and (b) more importance to the “site” variable than the other penalties. Note that there are differences in the proportion of males at each study site; thus, the “sex” and “site” variables contain shared information (i.e., dependence), which may explain the noteworthy difference in the variable importances attributed to these terms in the compared models). The “income” variable also has a noteworthy importance in all three models: LASSO = 13.028, MCP = 11.363, and SCAD = 18.872. The parental history of problematic alcohol use (i.e., “alcohol”) has a rather small importance index for the LASSO and MCP, and is determined to be negligible in the SCAD model. Finally, the parental education variable (i.e., “edu”) is determined to be slightly important by the LASSO but unimportant in the other two models.

The LASSO model includes nine of the brain connectivity variables in the model, but several of the included variables have importance indices that are quite small (see Figure 4). The three most important rs-FC terms in the LASSO model have variable importance values of DT-DLA = 2.697, DT-CGC = 0.740, and CGC-VTA = 0.462. Interestingly, the MCP and SCAD models agree that the DT-DLA connection is the only important brain connectivity variable in the model. The two models also agree regarding the importance of the DT-DLA connection, which has importance values of MCP = 11.052 and SCAD = 11.3082. As previously stated, these importance values give the approximate percentage of the overall model $R^{2}$ that is accounted for by each predictor, so the DT-DLA connection explains about $R_{\mathrm{MCP}}^{2}=1.042$% and $R_{\mathrm{SCAD}}^{2}=0.896$% of the CBCL Attention Problem scores—after controlling for the demographic and parental history variables.

### 3.3. Demographic and Parental History Effects

To understand the nature of the relationships estimated by the model, we first note that the assumed model has the form $\log\left(\mu \right)=\sum_{j=0}^{p}f_{j}$, where μ is the expected CBCL Attention Problems score, $f_{0}$ is an intercept term, and $f_{j}$ is the *j*-th predictor’s effect. Exponentiating both sides writes the model on the response scale, such as $\mu =\exp\left(\sum_{j=0}^{p}f_{j}\right)=\prod_{j=0}^{p}\exp\left(f_{j}\right)$. This implies that the exponential of the intercept, i.e., $\exp(f_{0})$, gives the baseline predicted CBCL Attention Problems score, which is $\exp(f_{0}^{\mathrm{LASSO}})=2.669$, $\exp(f_{0}^{\mathrm{MCP}})=1.452$, and $\exp(f_{0}^{\mathrm{SCAD}})=1.675$. Further, the exponential of the *j*-th effect, i.e., $\exp(f_{j})$, gives the multiplicative effect of the *j*-th predictor on the predicted response. It should be noted that, if $\exp(f_{j})>1$, then the *j*-th effect increases the baseline predicted CBCL Attention Problems score, whereas, if $\exp(f_{j})<1$, then the *j*-th effect decreases the baseline prediction.

Table 2 displays the multiplicative effects for the demographic and parental history variables, and Table 3 displays the multiplicative effects for the 22 different ABCD sites. The results in Table 2 are as expected based on the past literature: males are predicted to have more attention problems than females, having lower income increases the predicted attention problems, and having both parents with problematic histories of alcohol and drug use increases the predicted attention problems. Interestingly, if only one parent has an alcohol or drug problem, then the child would be expected to have more attention problems if the alcohol and/or drug problem is with the mother only (compared to the father only). The results in Table 3 reveal that there are noteworthy differences in the CBCL Attention Problem scores across the different ABCD sites, but the effect is much more pronounced in the MCP solution compared to the LASSO and SCAD solutions.

### 3.4. DT-DLA Functional Connectivity Effect

**Figure 5:**
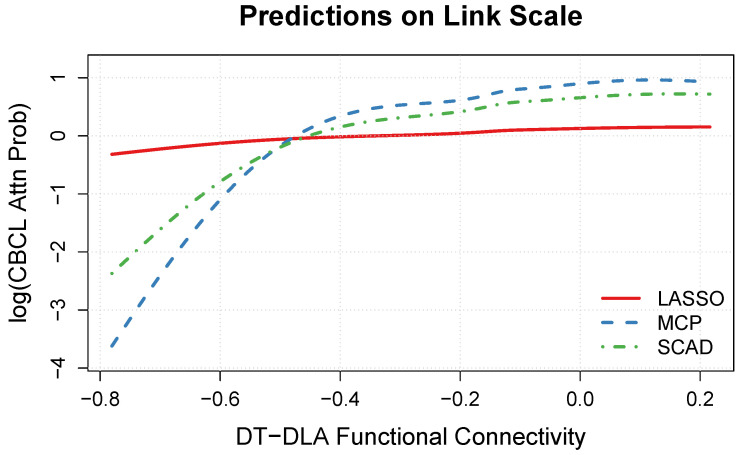
Predicted Child Behavior Checklist (CBCL) Attention Problems on the link scale (i.e., additive effect $f_{j}$) as a function of the resting-state functional connectivity between the default mode (DT) and the dorsal attention (DLA) networks.

**Figure 6:**
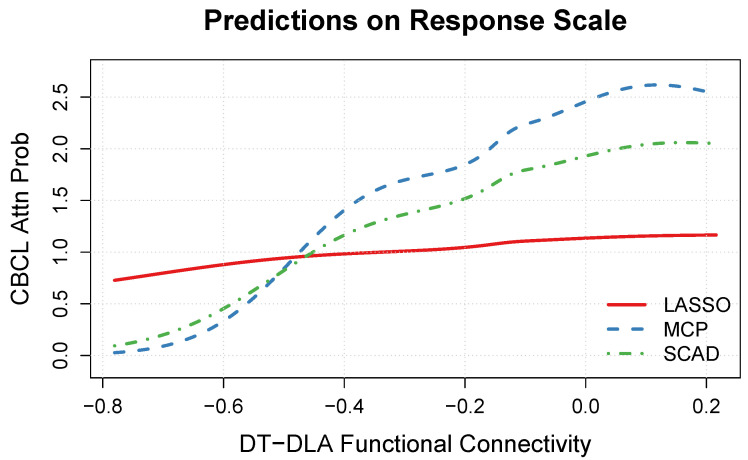
Predicted Child Behavior Checklist (CBCL) Attention Problems on the response scale (i.e., multiplicative effect $\exp(f_{j})$) as a function of the resting-state functional connectivity between the default mode (DT) and the dorsal attention (DLA) networks.

The estimated relationship between the DT-DLA connectivity and CBCL Attention Problems is plotted in Figure 5 and Figure 6. Focusing first on Figure 5, we note that the estimated relationship appears rather nonlinear in the MCP and SCAD solutions, but rather linear in the LASSO solution. More specifically, in the MCP and SCAD solutions, the logarithm of the predicted attention scores increases steeply as the magnitude of the connectivity decreases from −0.8 to −0.4, but then increases more gradually as the correlation approaches zero. In contrast, in the LASSO solution, the log of the predicted attention scores increases rather gradually across the observed range of connectivity values.

Next, focusing on Figure 6, we note that the estimated multiplicative effect appears much stronger in the MCP and SCAD solutions compared to the LASSO solution. More specifically, in the MCP and SCAD solutions, the multiplicative effect ranges from approximately 0 (DT-DLA connectivity = −0.8) to 2–2.5 (DT-DLA connectivity = 0.2), whereas the multiplicative effect in the LASSO solution ranges from approximately 0.7 to 1.2 across that same range of DT-DLA connectivity values. In the MCP and SCAD solutions, the multiplicative effect is less than one (which corresponds to reduced attention problems) when the DT-DLA connectivity is in the range −0.8 to −0.5 (strong negative correlation), whereas the multiplicative effect is greater than one (which corresponds to increased attention problems) when the DT-DLA connectivity is in the range −0.5 to 0.2 (weaker correlation).

## 4. Discussion

### 4.1. Summary of Findings

In this paper, we leveraged recent advances in penalized regression to explore the extent to which rs-FC predicts attention problems in children after controlling for demographic and parental history risk factors. Using a community sample of data from the ABCD study, we fit a penalized Poisson regression model to predict CBCL Attention Problem scores (see Figure 2) from 78 within- and between-network connections, as well as seven known risk factors (and study site). We compared three different variable selection penalties (LASSO, MCP, SCAD), and used 10-fold cross-validation to tune the model hyperparameters. For each penalty, we examined the nature of the cross-validation path, the importance attributed to each model term, and the nature of the estimated functional relationship between the CBCL Attention Problem scores and each predictor. Code to reproduce all aspects of these analyses can be found in the Supplementary Material.

Our results reveal that the three different penalties produced similar predictive performance in terms of the MAE (see Figure 3), but the different penalties produced rather noteworthy differences in the importance assigned to each model term (see Figure 4). All three penalties agreed that parental history of problematic drug use was the most influential predictor, followed by the biological sex of the child. Examination of the estimated effects in Table 2 reveals that our results agree with past literature on the topic regarding the nature of the drug use (see [16]) and sex effects (see [2]); specifically, parental history of problematic drug usage and being a male both relate to increased attention problems. The three penalties also agree that having lower income increases the expected attention problems in children, which is consistent with the past literature (see [14]).

Of primary interest in our study was the estimated relationships between the rs-FC measures and the CBCL Attention Problems scores. Of the 78 within- and between-network connections, the MCP and SCAD penalties only selected a single feature, which was the connection between the default mode network (DT) and the dorsal attention network (DLA). It should be noted that the LASSO penalty selected the DT-DLA connection, as well as eight additional connections, but the LASSO penalty attributed the largest importance to the DT-DLA connection. For the LASSO penalty to provide consistent variable selection, the data need to meet rather strict assumptions (see [56,61]); consequently, we prefer the MCP and SCAD solutions, given that these penalties are capable of consistent variable selection under a wider set of circumstances (see [55]).

Focusing on the MCP and SCAD solutions, the model predictions reveal that there is a noteworthy nonlinear relationship between the strength of the DT-DLA connection and the CBCL Attention Problems scores (see Figure 5 and Figure 6). More specifically, a decrease in the magnitude of the connectivity between the default mode network (DT) and the dorsal attention network (DLA) corresponds to increased attention problems, as measured by the CBCL. Our primary finding complements and extends the recent results of Owens et al. [38], who found that ADHD symptomatology was associated with task-based fMRI performance, and Norman et al. [41], who found that ADHD diagnosis was associated with reduced anticorrelation between the DT and DLA networks. Thus, our results suggest that rs-FC may be useful for understanding individual differences in attention problems.

### 4.2. Strengths, Limitations, and Future Directions

The present study has numerous advantages that make it distinct from the existing literature: (i) we leveraged recent advances in elastic net regression to fit and tune our model using 10-fold cross-validation, (ii) we compared the influence of common and more advanced variable selection penalties to find an optimal solution, (iii) we used a dimensional measure of attention problems to understand individual differences in ADHD symptomatology, and (iv) we used the large, epidemiologically informed ABCD sample to ensure that our results are representative of children across the United States. Regarding points (i) and (ii), to the best of our knowledge, the present study is the first attempt to use a nonparametric elastic net approach in combination with advanced variable selection penalties to predict behavior from neuroimaging data. Regarding points (iii) and (iv), the present study is the first study that attempts to predict a dimensional measure of ADHD symptomatology from rs-FC using data from the ABCD study.

Despite these obvious advantages, there are some subtle limitations of the present study that could be improved upon in future efforts. One such limitation is that the ABCD sample contains siblings, and this family structure was not accounted for in the current analyses—although previous research has shown that this is unlikely to have a meaningful effect [38,62]. Our choice to use a dimensional measure of ADHD symptomatology, which we view as a strength, could also be considered a limitation of our study. Research suggests that dimensional measures of psychopathology have greater reliability and validity [63], and previous genetic [64] and neurocognitive [65] work supports a dimensional view of ADHD in particular. However, in practice, ADHD is treated as a categorical diagnosis, and recent work has suggested that using a restrictive, multi-informant classification of ADHD may lead to stronger associations with genetic and cognitive signals of interest [66]. Finally, there is always difficulty in using fMRI to investigate children with ADHD, and functional connectivity in particular is highly sensitive to artifacts from motion [67]. We retained as many subjects as possible while maintaining data quality; however, it is possible that subjects with greater ADHD symptomatology or who were otherwise more susceptible to motion were more likely to be removed from the sample, limiting generalizability [68], or that motion differences are contributing to findings.

## 5. Conclusions

In summary, our findings demonstrate the potential predictive power of resting-state functional connectivity information in youth with ADHD. As one of the largest investigations to date of the rs-FC networks associated with ADHD in children, our finding of the importance of the DT-DLA correlation coheres with other large-scale studies using differing approaches and datasets [41], helping to begin to establish a consensus in an area of research marred by inconsistent findings [36,37]. As the field moves more towards embracing large-scale consortia studies, data-driven approaches for identifying relevant predictors will become increasingly common, but careful attention must be paid to what variable selection strategies are used and how modeling choices (such as the penalty type) affect the subsequent conclusions. We hope that our data-driven approach establishes directions for future research, including replication of and further investigation into the DT-DLA connection highlighted in the present work as it relates to ADHD, as well as similar variable selection approaches for data from other sources and relating to other clinical disorders.

## Figures and Tables

**Table 1:** Descriptive statistics of demographic variables for the sample used in analyses (*N* = 7979).

Variable	Factor Levels	Percentage (%)
Sex	Male	49.994
	Female	50.006
Race/Ethnicity	White	56.937
	Black/African-American	12.094
	Native American/Alaska Native	0.251
	Asian/Pacific Islander	1.466
	Hispanic/Latino	19.539
	Multiple Races/Other	9.713
Household Income	<50 k	27.334
	50–100 k	28.688
	>100 k	43.978
Parental Education	HS Diploma or Less	11.342
	Some College	25.116
	Bachelor’s	26.983
	Graduate	36.559
Parental Alcohol Use	None	85.061
	Father Only	11.067
	Mother Only	2.231
	Both Parents	1.642
Parental Drug Use	None	69.207
	Father Only	8.059
	Mother Only	16.155
	Both Parents	6.580

**Table 2:** Estimated multiplicative effects for each active demographic/parental history variable.

Variable	Factor Levels	LASSO	MCP	SCAD
Sex	Male	1.15	1.17	1.18
	Female	0.87	0.85	0.85
Income	<50 k	1.10	1.13	1.16
	50–100 k	0.99	0.99	0.99
	>100 k	0.92	0.89	0.87
Alcohol	None	0.94	0.87	0.98
	Father Only	0.96	0.93	0.99
	Mother Only	1.00	1.03	1.00
	Both Parents	1.11	1.19	1.04
Drugs	None	0.79	0.76	0.75
	Father Only	0.93	0.93	0.93
	Mother Only	1.10	1.12	1.12
	Both Parents	1.24	1.27	1.29

**Table 3:** Estimated multiplicative effect for each site in the ABCD study.

Site	LASSO	MCP	SCAD
site01	0.93	0.77	0.98
site02	0.94	0.81	0.98
site03	1.09	1.37	1.03
site04	1.05	1.17	1.01
site05	1.01	1.06	1.00
site06	0.99	0.97	1.00
site07	1.04	1.15	1.01
site08	1.02	1.09	1.01
site09	0.97	0.95	0.99
site10	0.94	0.79	0.98
site11	1.04	1.14	1.01
site12	1.04	1.13	1.01
site13	0.97	0.90	0.99
site14	0.94	0.80	0.98
site15	1.04	1.12	1.01
site16	1.02	1.13	1.01
site17	0.99	0.96	1.00
site18	0.98	0.91	0.99
site19	0.96	0.86	0.99
site20	1.00	1.04	1.00
site21	1.01	1.05	1.00
site22	1.03	1.08	1.01

## Data Availability

R code to reproduce our results is included as supplementary materials. Data used in the preparation of this article were obtained from the Adolescent Brain Cognitive Development (ABCD) Study (https://abcdstudy.org), held in the NIMH Data Archive (NDA). The ABCD data repository grows and changes over time. The ABCD data used in this report came from Data Release Version 3.0 found at http://dx.doi.org/10.15154/1520591.

## Supplementary Materials

The following supporting information can be downloaded at https://www.mdpi.com/article/10.3390/neurosci5040033/s1: Code: cbcl-fmri-supmat.html.

## Abbreviations

Brain network abbreviations:

AD	Auditory network
CA	Cingulo-parietal network
CGC	Cingulo-opercular network
DLA	Dorsal attention network
DT	Default network
FO	Fronto-parietal network
RSPLTP	Retrosplenial temporal network
SA	Salience network
SMH	Somatomotor hand network
SMM	Somatomotor mouth network
VIS	Visual network
VTA	Ventral attention network

Other Abbreviations:

ABCD	Adolescent Brain Cognitive Development Study
ADHD	Attention-deficit hyperactivity disorder
CBCL	Child Behavior Checklist
fMRI	Functional magnetic resonance imaging
LASSO	Least absolute shrinkage and selection operator
MAE	Mean absolute error
MCP	Minimax concave penalty
QC	Quality control
ROIs	Regions of interest
rs-FC	Resting-state functional connectivity
rs-fMRI	Resting-state fMRI
SCAD	Smoothly clipped absolute deviation

## Appendix A

Following collection, the rs-fMRI data were processed and QC was performed by the ABCD Data Analysis and Informatics Center (DAIC) in accordance with the study protocol. For processing, initial frames were removed, and voxel time series were normalized by voxel mean. Then linear regression removed signals correlated with estimated motion time series, quadratic trends, and the mean time courses and first derivatives of white matter, ventricles, and the whole brain.

Motion regression was performed using six parameters of motion along with their squares and derivatives on all frames with <0.30 mm framewise displacement (FD), and temporal filtering was used on the estimated motion time series to mitigate respiratory signal. Time series were then bandpass-filtered between 0.009 and 0.08 Hz. The preprocessed time series were then sampled onto each individual subject’s cortical surface, and average region of interest (ROI) time series were calculated for the functionally defined Gordon atlas parcellation [42], which are resampled from atlas space into subject space.

Automated and manual QC were performed by the DAIC in accordance with the study protocol [45]. Briefly, automated QC procedures included checking for completeness of files, adherence to protocol, and calculation of metrics such as mean FD and number of time points below various thresholds of FD. Manual QC procedures included reviewing images for artifacts and irregularities. Any subjects that were determined to have failed the rs-fMRI QC performed by the DAIC were removed from the analysis.
