# Supplementary material for: Resting-State Functional Connectivity Predicts Attention Problems in Children: Evidence from the ABCD Study
Source: NeuroSci. 2024 Oct 12;5(4):445–61. doi: 10.3390/neurosci5040033 (PMC11503400; doi:10.3390/neurosci5040033)
Supplement: Supplementary file 1 [file neurosci-05-00033-s001.zip › neurosci-3209950-supplementary.html]

Resting-State Functional Connectivity Predicts Attention Problems in Children: Evidence from the ABCD Study


# Resting-State Functional Connectivity Predicts Attention Problems in Children: Evidence from the ABCD Study

#### Kelly A. Duffy and Nathaniel E. Helwig

#### Online Supplement (R Code) Submitted to NeuroSci (2024-10-03)

- Install and Load grpnet
  Package
- Merge ABCD
  Data
- Load ABCD
  Data
- Preprocess ABCD Data
- Figure 2: CBCL Attention
  Problems
- Table 1: Descriptive
  Statistics
- Fit and
  Tune Models
- Model R-squared
  (Explained Poisson Deviance)
- Figure 3:
  Cross-Validation Error Paths
- Figure 4: Variable
  Importance Indices
- Estimated Baseline
  CBCL Attenion Problems
- Table 2:
  Estimated Demographic/Parental Effects
- Table 3: Estimated Study
  Site Effect
- Figure 5:
  Estimated DT-DLA Connectivity Effect

# Install and Load grpnet Package

Install and load the **grpnet**
package (version 0.5)

```
if(!require(grpnet)) install.packages("grpnet"); library(grpnet)
```

```
## Loading required package: grpnet
```

```
## Package 'grpnet' version 0.5
## Type 'citation("grpnet")' to cite this package.
```

Define cluster for parallel computing

```
cl <- parallel::makeCluster(min(10, parallel::detectCores()))
```

# Merge ABCD Data

**CBCL Data**

```
# Load in data from 'abcd_cbcls01' file
cbcl.names <- read.table('rawdata/abcd_cbcls01.txt', nrow = 1)
cbcl <- read.table('rawdata/abcd_cbcls01.txt', skip = 2)
colnames(cbcl) <- as.character(cbcl.names[1,])
rm(cbcl.names)

# Separate out baseline data
cbcl.baseline <- cbcl[which(cbcl$eventname=="baseline_year_1_arm_1"),
                      c("src_subject_id","cbcl_scr_dsm5_adhd_r")]
```

**Study Site**

```
# Load in longitudinal tracking data from 'abcd_lt01' file
long.names <- read.table('rawdata/abcd_lt01.txt', nrow = 1)
long <- read.table('rawdata/abcd_lt01.txt', skip = 2)
colnames(long) <- as.character(long.names[1,])
rm(long.names)

# Separate out baseline data
long.baseline <- long[which(long$eventname == "baseline_year_1_arm_1"),
                      c("src_subject_id","site_id_l")]
```

**Demographics: Sex**

```
# Load in parent demographic survey data from 'pdem02' file
dem.names <- read.table('rawdata/pdem02.txt', nrow = 1)
dem <- read.table('rawdata/pdem02.txt', skip = 2)
colnames(dem) <- as.character(dem.names[1,])
rm(dem.names)

# Get sex assigned at birth
dem$sex <- as.numeric(dem$demo_sex_v2)
dem$sex[dem$sex == 3] <- 1        # recode 4 intersex males as male
```

**Demographics: Race**

```
# If demo_race_a_p___10 is 1, set as level 1 (white)
dem$demo_race_eth[as.numeric(dem$demo_race_a_p___10) == 1] <- 1

# If demo_race_a_p___11 is 1, set as level 2 (Black/African-American)
dem$demo_race_eth[as.numeric(dem$demo_race_a_p___11) == 1] <- 2

# If demo_race_a_p___12 OR demo_race_a_p___13 is 1, set as level 3 (Native American/American Indian/Alaskan Native) 
dem$demo_race_eth[as.numeric(dem$demo_race_a_p___12) == 1] <- 3
dem$demo_race_eth[as.numeric(dem$demo_race_a_p___13) == 1] <- 3

# If demo_race_a_p___14 THROUGH demo_race_a_p___24 is 1, set as level 4 (Asian/Pacific Islander) 
dem$demo_race_eth[as.numeric(dem$demo_race_a_p___14) == 1] <- 4
dem$demo_race_eth[as.numeric(dem$demo_race_a_p___15) == 1] <- 4
dem$demo_race_eth[as.numeric(dem$demo_race_a_p___16) == 1] <- 4
dem$demo_race_eth[as.numeric(dem$demo_race_a_p___17) == 1] <- 4
dem$demo_race_eth[as.numeric(dem$demo_race_a_p___18) == 1] <- 4
dem$demo_race_eth[as.numeric(dem$demo_race_a_p___19) == 1] <- 4
dem$demo_race_eth[as.numeric(dem$demo_race_a_p___20) == 1] <- 4
dem$demo_race_eth[as.numeric(dem$demo_race_a_p___21) == 1] <- 4
dem$demo_race_eth[as.numeric(dem$demo_race_a_p___22) == 1] <- 4
dem$demo_race_eth[as.numeric(dem$demo_race_a_p___23) == 1] <- 4
dem$demo_race_eth[as.numeric(dem$demo_race_a_p___24) == 1] <- 4

# If demo_race_a_p___25  is 1, set as level 6 ("Other"/NA, as specified by parent)
dem$demo_race_eth[as.numeric(dem$demo_race_a_p___25) == 1] <- 6

# If multiple specified, add into 6 category
dem[,c(17:32)] <- sapply(dem[,c(17:32)],as.numeric)
dem$mixed_race_eth <- rowSums(dem[, c(17:32)], na.rm=TRUE)
dem$demo_race_eth[dem$mixed_race_eth > 1] <- 6

# Specify level 5 if they indicate they are Hispanic/Latino
dem$demo_race_eth[as.numeric(dem$demo_ethn_v2) == 1] <- 5

# Rename var
dem$race <- dem$demo_race_eth
```

**Demographics: Income**

```
# Get household income
dem$income <- as.numeric(dem$demo_comb_income_v2)
dem$income[dem$income == 777] <- NA      # recode missing
dem$income[dem$income == 999] <- NA      # recode not reported

# Code 1-6 as <50k (1)
dem$income[dem$income <= 6] <- 1

# Code 7 and 8 as 50-100k (2)
dem$income[dem$income == 7] <- 2
dem$income[dem$income == 8] <- 2

# Code 9 and 10 as > 100k (3)
dem$income[dem$income == 9] <- 3
dem$income[dem$income == 10] <- 3
```

**Demographics: Parent Education**

Education for Parent 1

```
# Start with parent 1 (parent filling out questionnaire)
dem$parent1.edu <- as.numeric(dem$demo_prnt_ed_v2)
dem$parent1.edu[dem$parent1.edu == 777] <- NA      # recode missing
dem$parent1.edu[dem$parent1.edu == 999] <- NA      # recode not reported

# Re-code 1-14 as HS (or equivalent GED)
dem$parent1.edu[dem$parent1.edu <= 14] <- 1

# Score 15, 16, and 17 as some college (but really includes associate's) 
dem$parent1.edu[dem$parent1.edu == 15] <- 2
dem$parent1.edu[dem$parent1.edu == 16] <- 2
dem$parent1.edu[dem$parent1.edu == 17] <- 2

# Re score 18 as bachelor's (3)
dem$parent1.edu[dem$parent1.edu == 18] <- 3

# Score 19, 20 and 21 as graduate
dem$parent1.edu[dem$parent1.edu == 19] <- 4
dem$parent1.edu[dem$parent1.edu == 20] <- 4
dem$parent1.edu[dem$parent1.edu == 21] <- 4
```

Education for Parent 2

```
# Now do partner/person who helps raise child
dem$parent2.edu <- as.numeric(dem$demo_prtnr_ed_v2)
dem$parent2.edu[dem$parent2.edu == 777] <- NA      # recode missing
dem$parent2.edu[dem$parent2.edu == 999] <- NA      # recode not reported

# Score 1-14 as HS (or equivalent GED)
dem$parent2.edu[dem$parent2.edu <= 14] <- 1

# Score 15, 16, and 17 as some college (but really includes associate's) 
dem$parent2.edu[dem$parent2.edu == 15] <- 2
dem$parent2.edu[dem$parent2.edu == 16] <- 2
dem$parent2.edu[dem$parent2.edu == 17] <- 2

# Re score 18 as bachelor's (3)
dem$parent2.edu[dem$parent2.edu == 18] <- 3

# Score 19, 20 and 21 as graduate
dem$parent2.edu[dem$parent2.edu == 19] <- 4
dem$parent2.edu[dem$parent2.edu == 20] <- 4
dem$parent2.edu[dem$parent2.edu == 21] <- 4
```

Maximum Parent Education

```
# Set NA values to 0 before using pmax
dem$parent1.edu[is.na(dem$parent1.edu)] <- 0
dem$parent2.edu[is.na(dem$parent2.edu)] <- 0

# Run pmax to get max edu
dem$edu <- pmax(dem$parent1.edu, dem$parent2.edu)

# Set 0 values back to NA
dem$edu[dem$edu == 0] <- NA
```

**Family History: Parent Alcohol**

```
# Load in data from 'abcd_fhxssp01' file
famhist.names <- read.table('rawdata/abcd_fhxssp01.txt', nrow = 1)
fam.hist <- read.table('rawdata/abcd_fhxssp01.txt', skip = 2)
colnames(fam.hist) <- as.character(famhist.names[1,])
rm(famhist.names)

# Create parental history of problematic alcohol use variable, recoding some of the options for one parent affected
fam.hist$alcohol <- as.numeric(fam.hist$famhx_ss_parent_alc_p)

# Recode mother missing, father positive as 1 (father only)
fam.hist$alcohol[fam.hist$alcohol == -2] <- 1

# Recode father missing, mother positive as 2 (mother only)
fam.hist$alcohol[fam.hist$alcohol == -1] <- 2
```

**Family History: Parent Drugs**

```
# Create parental history of problematic drug use variable
fam.hist$drugs <- as.numeric(fam.hist$famhx_ss_parent_dg_p)

# Recode mother missing, father positive as 1 (father only)
fam.hist$drugs[fam.hist$drugs == -2] <- 1

# Recode father missing, mother positive as 2 (mother only)
fam.hist$drugs[fam.hist$drugs == -1] <- 2
```

**Extract Demographics and Family
History**

```
# final demographics
dem <- dem[,c("src_subject_id","sex","race", "income","edu")]

# final family history
fam.hist <- fam.hist[,c("src_subject_id","alcohol","drugs")]
```

**Brain Connectivity**

```
# Load in data from 'abcd_betnet02' file
brain.net.corrs.names <- read.table('rawdata/abcd_betnet02.txt', nrow = 1)
brain.net.corrs <- read.table('rawdata/abcd_betnet02.txt', skip = 2)
colnames(brain.net.corrs) <- as.character(brain.net.corrs.names[1,])
rm(brain.net.corrs.names)

# subset baseline data
brain.net.baseline <- subset(brain.net.corrs, eventname == "baseline_year_1_arm_1")

# Create age variable
brain.net.baseline$age <- as.numeric(brain.net.baseline$interview_age) / 12

# age dataframe
age.baseline <- brain.net.baseline[,c("src_subject_id", "age")]

# column and network
cnames <- colnames(brain.net.baseline)
networks <- c("vta", "cgc", "sa", "dla", "dt", "ca", "fo", "rspltp", "ad", "vs", "smh", "smm")

# extract relevant network correlations
brainid <- c("src_subject_id", grep(paste(networks, collapse = "|"), cnames, value = TRUE))
net.corrs.only <- brain.net.baseline[,brainid]

# remove those with "_n_" in middle or "_n" at end, which correspond to the "none" network
noneid <- grep("_n_|_n$", names(net.corrs.only))
net.corrs.only <- net.corrs.only[,-noneid]
```

**Imaging Quality Control**

```
# Load in data from 'abcd_imgincl01' file
img.qc.names <- read.table('rawdata/abcd_imgincl01.txt', nrow = 1)
img.qc <- read.table('rawdata/abcd_imgincl01.txt', skip = 2)
colnames(img.qc) <- as.character(img.qc.names[1,])
rm(img.qc.names)

# Separate out baseline data
img.qc.time1 <- img.qc[which(img.qc$eventname=="baseline_year_1_arm_1"),
                       c("src_subject_id","imgincl_rsfmri_include")]
```

**Merge and Remove Bad Data**

```
# merge cbcl and site
abcd <- merge(cbcl.baseline, long.baseline, by = "src_subject_id")

# merge with age
abcd <- merge(abcd, age.baseline, by = "src_subject_id")

# merge with demographics
abcd <- merge(abcd, dem, by = "src_subject_id")

# merge with family history
abcd <- merge(abcd, fam.hist, by = "src_subject_id")

# merge with brain connectivity
abcd <- merge(abcd, net.corrs.only, by = "src_subject_id")

# merge with image QC
abcd <- merge(abcd, img.qc.time1, by = "src_subject_id")

# set blank cells to NA 
abcd[abcd == ""] <- NA

# remove those who didn't pass image QC check
abcd <- abcd[which(abcd$imgincl_rsfmri_include == 1),]

# remove those with missing data on relevant measures
abcd <- na.omit(abcd)
```

**Simplify Brain Network Names**

```
# extract column names
cnames <- names(abcd)

# simplify cbcl
id <- which(cnames == "cbcl_scr_dsm5_adhd_r")
cnames[id] <- "cbcl"

# simplify site
id <- which(cnames == "site_id_l")
cnames[id] <- "site"

# simplify brain network connectivity names
cnames <- gsub("rsfmri_c_ngd_", "", cnames) 
cnames <- gsub("_ngd_", "_", cnames) 

# rename columns
names(abcd) <- cnames
```

**Reorder Columns and Save**

```
# brain network names
networks <- c("vta", "cgc", "sa", "dla", "dt", "ca", "fo", "rspltp", "ad", "vs", "smh", "smm")
numnet <- length(networks)

# all unique network pairings
unipairs <- NULL
for(j in 1:numnet){
  for(k in 1:j){
    unipairs <- c(unipairs, paste(networks[j], networks[k], sep = "_"))
  }
}

# character column
abcd$site <- as.character(abcd$site)

# integer columns
intid <- c("cbcl", "sex", "race", "income", "edu", "alcohol", "drugs")
for(i in intid){
  abcd[,i] <- as.integer(abcd[,i])
}

# numeric columns
numid <- c("age", unipairs)
for(i in numid){
  abcd[,i] <- as.numeric(abcd[,i])
}

# define variable names to save
savenames <- c("cbcl", "site", "age", "sex", "race", "income", 
               "edu", "alcohol", "drugs", unipairs)

# extract desired variables
abcd <- abcd[,savenames]

# save as csv file
write.csv(abcd, file = "abcd.csv", row.names = FALSE)

# remove junk
junk <- c(ls(), "junk")
id <- which(junk %in% c("abcd", "cl"))
rm(list = junk[-id])
```

# Load ABCD Data

Load ‘abcd’ data

```
abcd <- read.csv("abcd.csv")
```

Check ‘abcd’ data dimensions

```
dim(abcd)
```

```
## [1] 7979   87
```

Check ‘abcd’ data structure

```
str(abcd)
```

```
## 'data.frame':    7979 obs. of  87 variables:
##  $ cbcl         : int  0 4 5 7 1 2 0 14 8 2 ...
##  $ site         : chr  "site06" "site22" "site07" "site20" ...
##  $ age          : num  10.92 10.5 9.33 10.83 9.08 ...
##  $ sex          : int  2 1 1 1 1 2 2 2 1 2 ...
##  $ race         : int  1 1 1 1 5 1 1 3 1 5 ...
##  $ income       : int  2 3 3 1 1 2 3 1 3 1 ...
##  $ edu          : int  1 4 4 2 1 3 4 1 3 2 ...
##  $ alcohol      : int  0 0 0 0 1 0 0 0 0 0 ...
##  $ drugs        : int  2 1 2 1 0 3 2 3 1 0 ...
##  $ vta_vta      : num  0.31 0.24 0.182 0.196 0.219 ...
##  $ cgc_vta      : num  0.01705 0.02346 0.08146 0.00582 0.05816 ...
##  $ cgc_cgc      : num  0.382 0.36 0.244 0.228 0.363 ...
##  $ sa_vta       : num  0.1221 0.0106 0.1285 0.0468 0.0701 ...
##  $ sa_cgc       : num  0.0315 0.147 0.1218 0.1149 0.1659 ...
##  $ sa_sa        : num  0.411 0.309 0.369 0.3 0.524 ...
##  $ dla_vta      : num  -0.15022 -0.09602 -0.00685 0.00226 -0.05926 ...
##  $ dla_cgc      : num  0.1194 0.0547 0.1118 0.0959 0.0464 ...
##  $ dla_sa       : num  -0.02181 -0.00603 0.00932 0.03662 -0.0144 ...
##  $ dla_dla      : num  0.315 0.24 0.24 0.194 0.227 ...
##  $ dt_vta       : num  0.1389 0.0566 0.016 0.068 0.035 ...
##  $ dt_cgc       : num  -0.2082 -0.188 -0.0843 -0.09 -0.1342 ...
##  $ dt_sa        : num  0.08105 -0.00397 0.08285 0.09856 0.03122 ...
##  $ dt_dla       : num  -0.2344 -0.0736 -0.0894 -0.0744 -0.124 ...
##  $ dt_dt        : num  0.339 0.256 0.267 0.207 0.212 ...
##  $ ca_vta       : num  -0.2655 -0.1881 -0.0729 -0.0579 -0.1095 ...
##  $ ca_cgc       : num  -0.0237 0.0187 -0.0856 0.0318 0.0472 ...
##  $ ca_sa        : num  0.0748 0.1458 -0.0845 0.2096 0.2871 ...
##  $ ca_dla       : num  0.0903 0.0632 -0.125 0.1277 -0.0583 ...
##  $ ca_dt        : num  -0.0447 0.0156 0.1211 -0.0511 0.059 ...
##  $ ca_ca        : num  1.128 0.935 0.743 0.882 0.83 ...
##  $ fo_vta       : num  0.04217 0.01131 0.1209 -0.00813 -0.03869 ...
##  $ fo_cgc       : num  -0.0263 0.0391 0.0308 -0.0156 0.0116 ...
##  $ fo_sa        : num  0.1704 0.1154 0.0905 0.175 0.1073 ...
##  $ fo_dla       : num  0.0516 0.0741 0.0398 0.0519 0.0419 ...
##  $ fo_dt        : num  0.0115 -0.0181 0.0457 0.0961 0.0163 ...
##  $ fo_ca        : num  0.067 0.0658 -0.0279 0.0262 0.0819 ...
##  $ fo_fo        : num  0.198 0.249 0.214 0.301 0.238 ...
##  $ rspltp_vta   : num  -0.205 -0.118 -0.07 -0.127 -0.113 ...
##  $ rspltp_cgc   : num  -0.12811 -0.2221 -0.11418 -0.00802 -0.11935 ...
##  $ rspltp_sa    : num  -0.1142 -0.1497 0.0198 -0.0662 -0.1273 ...
##  $ rspltp_dla   : num  0.0878 0.0282 -0.0417 -0.0292 -0.0102 ...
##  $ rspltp_dt    : num  0.025 0.1408 0.1285 -0.0354 0.0341 ...
##  $ rspltp_ca    : num  0.344 0.152 0.268 0.214 0.155 ...
##  $ rspltp_fo    : num  -0.0614 -0.1428 -0.0377 -0.127 -0.1403 ...
##  $ rspltp_rspltp: num  0.725 0.785 0.385 0.413 0.645 ...
##  $ ad_vta       : num  0.0738 0.0787 0.0762 0.0683 0.1262 ...
##  $ ad_cgc       : num  0.2235 0.2074 0.1088 0.1755 0.0954 ...
##  $ ad_sa        : num  -0.00354 0.09335 0.07386 -0.02612 -0.09786 ...
##  $ ad_dla       : num  0.00508 -0.0547 0.00211 -0.02618 -0.05694 ...
##  $ ad_dt        : num  -0.0936 -0.1236 -0.0318 -0.1168 -0.0638 ...
##  $ ad_ca        : num  -0.1255 -0.0892 -0.0895 -0.0691 -0.1662 ...
##  $ ad_fo        : num  -0.0501 0.0374 -0.0185 -0.1358 -0.1074 ...
##  $ ad_rspltp    : num  -0.0813 -0.1796 -0.1194 0.0143 0.0116 ...
##  $ ad_ad        : num  0.435 0.288 0.192 0.374 0.424 ...
##  $ vs_vta       : num  -0.1956 -0.0811 -0.0958 -0.0752 -0.1093 ...
##  $ vs_cgc       : num  -0.0537 0.0186 -0.0331 0.0371 0.0177 ...
##  $ vs_sa        : num  -0.09307 -0.00353 -0.11939 -0.09657 -0.09333 ...
##  $ vs_dla       : num  0.0942 -0.0262 -0.0256 0.0347 0.0334 ...
##  $ vs_dt        : num  -0.0913 -0.1462 -0.1083 -0.1924 -0.1652 ...
##  $ vs_ca        : num  0.2583 0.0795 0.0776 0.2508 0.0388 ...
##  $ vs_fo        : num  -0.0417 -0.1081 -0.0991 -0.2612 -0.1628 ...
##  $ vs_rspltp    : num  0.25042 -0.00243 0.0667 0.26662 0.26851 ...
##  $ vs_ad        : num  -0.0991 -0.011 -0.0567 0.0933 0.0285 ...
##  $ vs_vs        : num  0.368 0.435 0.282 0.579 0.538 ...
##  $ smh_vta      : num  -0.08165 0.00213 -0.01829 0.02895 0.04554 ...
##  $ smh_cgc      : num  0.1461 0.1486 0.0659 0.0818 -0.0585 ...
##  $ smh_sa       : num  -0.11608 -0.00476 0.06986 -0.10531 -0.179 ...
##  $ smh_dla      : num  0.10992 0.06318 0.04995 0.00239 0.02728 ...
##  $ smh_dt       : num  -0.1308 -0.0787 -0.0708 -0.1342 -0.0355 ...
##  $ smh_ca       : num  -0.0759 -0.1418 -0.1123 -0.0789 -0.2272 ...
##  $ smh_fo       : num  -0.06112 -0.00755 -0.07881 -0.11276 -0.14347 ...
##  $ smh_rspltp   : num  -0.00297 -0.09915 -0.04179 -0.08843 0.04758 ...
##  $ smh_ad       : num  0.1579 0.0961 0.1086 0.2772 0.2515 ...
##  $ smh_vs       : num  -0.0209 -0.0597 -0.0634 0.0193 0.0177 ...
##  $ smh_smh      : num  0.295 0.293 0.31 0.391 0.393 ...
##  $ smm_vta      : num  -0.0212 0.0302 -0.0257 0.0943 -0.0102 ...
##  $ smm_cgc      : num  0.1282 0.2725 0.0409 0.0841 0.0248 ...
##  $ smm_sa       : num  0.1018 0.0318 -0.1002 -0.1475 -0.1369 ...
##  $ smm_dla      : num  0.0496 -0.0901 0.0136 -0.0408 -0.056 ...
##  $ smm_dt       : num  -0.0818 -0.1914 -0.0781 -0.1231 -0.077 ...
##  $ smm_ca       : num  -0.0671 -0.1111 -0.0837 -0.1099 -0.0701 ...
##  $ smm_fo       : num  -0.0334 -0.0394 -0.0639 -0.2185 -0.151 ...
##  $ smm_rspltp   : num  -0.0229 -0.2278 -0.0848 0.0253 0.1001 ...
##  $ smm_ad       : num  0.234 0.288 0.144 0.415 0.249 ...
##  $ smm_vs       : num  0.05131 0.09708 -0.00308 0.12658 0.17823 ...
##  $ smm_smh      : num  0.0617 0.0997 0.0796 0.3422 0.1855 ...
##  $ smm_smm      : num  0.595 0.761 0.431 0.712 0.615 ...
```

# Preprocess ABCD Data

Convert site to unordered factor

```
abcd$site <- as.factor(abcd$site)
```

Convert sex to unordered factor

```
abcd$sex <- factor(abcd$sex, levels = 1:2, labels = c("male", "female"))
```

Convert race to unordered factor

```
abcd$race <- factor(abcd$race, levels = 1:6, 
                    labels = c("White", "Black", "Native", "Asian", "Hispanic", "Other"))
```

Convert income to ordered factor

```
abcd$income <- factor(abcd$income, levels = 1:3, ordered = TRUE,
                      labels = c("<50k", "50-100k", ">100k"))
```

Convert education to ordered factor

```
abcd$edu <- factor(abcd$edu, levels = 1:4, ordered = TRUE,
                   labels = c("highschool", "college", "bachelors", "graduate"))
```

Convert alcohol to unordered factor

```
abcd$alcohol <- factor(abcd$alcohol, levels = 0:3,
                       labels = c("none", "father", "mother", "both"))
```

Convert drugs to unordered factor

```
abcd$drugs <- factor(abcd$drugs, levels = 0:3,
                     labels = c("none", "father", "mother", "both"))
```

# Figure 2: CBCL Attention Problems

```
# save pdf
pdf(file = "fig2_cbcl.pdf", width = 8, height = 5)
barplot(table(abcd$cbcl), ylim = c(0, 3000), xlab = "Score", ylab = "Frequency",
        main = "CBCL Attention Problems Distribution", cex.axis = 1.15,
        cex.lab = 1.25, cex.main = 1.5)
dev.off()
```

```
## quartz_off_screen 
##                 2
```

```
# print to html
barplot(table(abcd$cbcl), ylim = c(0, 3000), xlab = "Score", ylab = "Frequency",
        main = "CBCL Attention Problems Distribution", cex.axis = 1.15,
        cex.lab = 1.25, cex.main = 1.5)
```

# Table 1: Descriptive Statistics

Build table 1

```
tab1 <- NULL
for(term in c("sex", "race", "income", "edu", "alcohol", "drugs")){
  newperc <- 100 * table(abcd[,term]) / nrow(abcd)
  newdata <- data.frame(variable = term, 
                        levels = names(newperc),
                        percentage = round(as.numeric(newperc), 3))
  tab1 <- rbind(tab1, newdata)
}
rownames(tab1) <- 1:nrow(tab1)
write.csv(tab1, file = "tab1_demo.csv", row.names = FALSE)
```

Print table 1

```
knitr::kable(tab1)
```

| variable | levels | percentage |
| --- | --- | --- |
| sex | male | 49.994 |
| sex | female | 50.006 |
| race | White | 56.937 |
| race | Black | 12.094 |
| race | Native | 0.251 |
| race | Asian | 1.466 |
| race | Hispanic | 19.539 |
| race | Other | 9.713 |
| income | <50k | 27.334 |
| income | 50-100k | 28.688 |
| income | >100k | 43.978 |
| edu | highschool | 11.342 |
| edu | college | 25.116 |
| edu | bachelors | 26.983 |
| edu | graduate | 36.558 |
| alcohol | none | 85.061 |
| alcohol | father | 11.067 |
| alcohol | mother | 2.231 |
| alcohol | both | 1.642 |
| drugs | none | 69.207 |
| drugs | father | 8.059 |
| drugs | mother | 16.155 |
| drugs | both | 6.580 |

# Fit and Tune Models

Fit LASSO model

```
set.seed(1)
mod1 <- cv.grpnet(cbcl ~ ., data = abcd, family = "poisson",
                  penalty = "LASSO", parallel = TRUE, cl = cl)
mod1
```

```
## 
## Call:   cv.grpnet.formula(formula = cbcl ~ ., data = abcd, parallel = TRUE, 
##     cluster = cl, family = "poisson", penalty = "LASSO")
## 
## Measure:    Mean Absolute Error 
## 
##      Alpha  Lambda Index Measure      SE nzGroup nzCoef     Df
## min      1 0.02072    33   2.119 0.02190      64    319 231.83
## 1se      1 0.05766    22   2.140 0.02325      16     79  39.65
```

Fit MCP model

```
set.seed(1)
mod2 <- cv.grpnet(cbcl ~ ., data = abcd, family = "poisson",
                  penalty = "MCP", parallel = TRUE, cl = cl)
mod2
```

```
## 
## Call:   cv.grpnet.formula(formula = cbcl ~ ., data = abcd, parallel = TRUE, 
##     cluster = cl, family = "poisson", penalty = "MCP")
## 
## Measure:    Mean Absolute Error 
## 
##      Alpha  Lambda Index Measure      SE nzGroup nzCoef Df
## min      1 0.06328    21   2.117 0.02391      10     49 49
## 1se      1 0.07622    19   2.129 0.02396       7     36 36
```

Fit SCAD model

```
set.seed(1)
mod3 <- cv.grpnet(cbcl ~ ., data = abcd, family = "poisson",
                  penalty = "SCAD", parallel = TRUE, cl = cl)
mod3
```

```
## 
## Call:   cv.grpnet.formula(formula = cbcl ~ ., data = abcd, parallel = TRUE, 
##     cluster = cl, family = "poisson", penalty = "SCAD")
## 
## Measure:    Mean Absolute Error 
## 
##      Alpha  Lambda Index Measure      SE nzGroup nzCoef    Df
## min   0.75 0.07687    22   2.119 0.02383      14     69 45.63
## 1se   0.75 0.09259    20   2.136 0.02440       8     39 21.39
```

# Model R-squared (Explained Poisson Deviance)

R-squared for LASSO model

```
rsq1 <- mod1$grpnet.fit$dev.ratio[mod1$index]
rsq1
```

```
## [1] 0.12251056 0.08516147
```

R-squared for MCP model

```
rsq2 <- mod2$grpnet.fit$dev.ratio[mod2$index]
rsq2
```

```
## [1] 0.10197405 0.09429929
```

R-squared for SCAD model

```
rsq3 <- mod3$grpnet.fit$dev.ratio[mod3$index]
rsq3
```

```
## [1] 0.09839738 0.07927728
```

# Figure 3: Cross-Validation Error Paths

Scale lambda by alpha for mod3 (to align for plotting)

```
mod3$lambda <- mod3$lambda * mod3$grpnet.fit$alpha
mod3$lambda.min <- mod3$lambda.min * mod3$grpnet.fit$alpha
mod3$lambda.1se <- mod3$lambda.1se * mod3$grpnet.fit$alpha
```

Plot CV error path for LASSO, MCP, and SCAD

```
# save pdf
pdf(file = "fig3_cvm.pdf", width = 8, height = 8)
par(mfrow = c(3, 1), mar = c(4.5, 4.5, 4, 2) + 0.1)
plot(mod1, main = "Least Absolute Shrinkage and Selection Operator (LASSO)",
     xlim = c(-10, -1), ylim = c(2, 2.6),
     cex.axis = 1.25, cex.lab = 1.5, cex.main = 1.75)
plot(mod2, main = "Minimax Concave Penalty (MCP)",
     xlim = c(-10, -1), ylim = c(2, 2.6),
     cex.axis = 1.25, cex.lab = 1.5, cex.main = 1.75)
plot(mod3, main = "Smoothly Clipped Absolute Deviation (SCAD)",
     xlim = c(-10, -1), ylim = c(2, 2.6),
     cex.axis = 1.25, cex.lab = 1.5, cex.main = 1.75)
dev.off()
```

```
## quartz_off_screen 
##                 2
```

```
# print to html
par(mfrow = c(3, 1), mar = c(4.5, 4.5, 4, 2) + 0.1)
plot(mod1, main = "Least Absolute Shrinkage and Selection Operator (LASSO)",
     xlim = c(-10, -1), ylim = c(2, 2.6),
     cex.axis = 1.25, cex.lab = 1.5, cex.main = 1.75)
plot(mod2, main = "Minimax Concave Penalty (MCP)",
     xlim = c(-10, -1), ylim = c(2, 2.6),
     cex.axis = 1.25, cex.lab = 1.5, cex.main = 1.75)
plot(mod3, main = "Smoothly Clipped Absolute Deviation (SCAD)",
     xlim = c(-10, -1), ylim = c(2, 2.6),
     cex.axis = 1.25, cex.lab = 1.5, cex.main = 1.75)
```

Undo scale lambda by alpha for mod3 (to align for plotting)

```
mod3$lambda <- mod3$lambda / mod3$grpnet.fit$alpha
mod3$lambda.min <- mod3$lambda.min / mod3$grpnet.fit$alpha
mod3$lambda.1se <- mod3$lambda.1se / mod3$grpnet.fit$alpha
```

# Figure 4: Variable Importance Indices

Get importance indices via predict function

```
imp1 <- predict(mod1, newdata = abcd, type = "imp")
imp2 <- predict(mod2, newdata = abcd, type = "imp")
imp3 <- predict(mod3, newdata = abcd, type = "imp")
```

R-squared for terms (corresponding to lambda.1se solution)

```
100 * cbind(rsq1[2] * imp1, rsq2[2] * imp2, rsq3[2] * imp3)
```

```
##                       [,1]      [,2]        [,3]
## site          0.3327776131 1.9747720 0.034475225
## age           0.0000000000 0.0000000 0.000000000
## sex           2.6478897606 2.0958901 2.254375397
## race          0.0000000000 0.0000000 0.000000000
## income        1.1095069870 1.0715077 1.496094136
## edu           0.4194324113 0.0000000 0.005269146
## alcohol       0.2011725464 0.3599069 0.035381930
## drugs         3.4544536783 2.8856374 3.205652674
## vta_vta       0.0000000000 0.0000000 0.000000000
## cgc_vta       0.0393634909 0.0000000 0.000000000
## cgc_cgc       0.0000000000 0.0000000 0.000000000
## sa_vta        0.0000000000 0.0000000 0.000000000
## sa_cgc        0.0000000000 0.0000000 0.000000000
## sa_sa         0.0000000000 0.0000000 0.000000000
## dla_vta       0.0000000000 0.0000000 0.000000000
## dla_cgc       0.0000000000 0.0000000 0.000000000
## dla_sa        0.0000000000 0.0000000 0.000000000
## dla_dla       0.0000000000 0.0000000 0.000000000
## dt_vta        0.0000000000 0.0000000 0.000000000
## dt_cgc        0.0629927747 0.0000000 0.000000000
## dt_sa         0.0000000000 0.0000000 0.000000000
## dt_dla        0.2296450528 1.0422148 0.896479222
## dt_dt         0.0000000000 0.0000000 0.000000000
## ca_vta        0.0000000000 0.0000000 0.000000000
## ca_cgc        0.0000000000 0.0000000 0.000000000
## ca_sa         0.0000000000 0.0000000 0.000000000
## ca_dla        0.0000000000 0.0000000 0.000000000
## ca_dt         0.0000000000 0.0000000 0.000000000
## ca_ca         0.0000000000 0.0000000 0.000000000
## fo_vta        0.0000000000 0.0000000 0.000000000
## fo_cgc        0.0000000000 0.0000000 0.000000000
## fo_sa         0.0067578654 0.0000000 0.000000000
## fo_dla        0.0000000000 0.0000000 0.000000000
## fo_dt         0.0000000000 0.0000000 0.000000000
## fo_ca         0.0003419844 0.0000000 0.000000000
## fo_fo         0.0000000000 0.0000000 0.000000000
## rspltp_vta    0.0000000000 0.0000000 0.000000000
## rspltp_cgc    0.0000000000 0.0000000 0.000000000
## rspltp_sa     0.0000000000 0.0000000 0.000000000
## rspltp_dla    0.0000000000 0.0000000 0.000000000
## rspltp_dt     0.0000000000 0.0000000 0.000000000
## rspltp_ca     0.0000000000 0.0000000 0.000000000
## rspltp_fo     0.0006367539 0.0000000 0.000000000
## rspltp_rspltp 0.0000000000 0.0000000 0.000000000
## ad_vta        0.0000000000 0.0000000 0.000000000
## ad_cgc        0.0000000000 0.0000000 0.000000000
## ad_sa         0.0000000000 0.0000000 0.000000000
## ad_dla        0.0000000000 0.0000000 0.000000000
## ad_dt         0.0000000000 0.0000000 0.000000000
## ad_ca         0.0000000000 0.0000000 0.000000000
## ad_fo         0.0000000000 0.0000000 0.000000000
## ad_rspltp     0.0000000000 0.0000000 0.000000000
## ad_ad         0.0000000000 0.0000000 0.000000000
## vs_vta        0.0000000000 0.0000000 0.000000000
## vs_cgc        0.0000000000 0.0000000 0.000000000
## vs_sa         0.0000000000 0.0000000 0.000000000
## vs_dla        0.0000000000 0.0000000 0.000000000
## vs_dt         0.0000000000 0.0000000 0.000000000
## vs_ca         0.0006914090 0.0000000 0.000000000
## vs_fo         0.0000000000 0.0000000 0.000000000
## vs_rspltp     0.0000000000 0.0000000 0.000000000
## vs_ad         0.0000000000 0.0000000 0.000000000
## vs_vs         0.0000000000 0.0000000 0.000000000
## smh_vta       0.0000000000 0.0000000 0.000000000
## smh_cgc       0.0000000000 0.0000000 0.000000000
## smh_sa        0.0000000000 0.0000000 0.000000000
## smh_dla       0.0000000000 0.0000000 0.000000000
## smh_dt        0.0000000000 0.0000000 0.000000000
## smh_ca        0.0002191789 0.0000000 0.000000000
## smh_fo        0.0000000000 0.0000000 0.000000000
## smh_rspltp    0.0000000000 0.0000000 0.000000000
## smh_ad        0.0000000000 0.0000000 0.000000000
## smh_vs        0.0000000000 0.0000000 0.000000000
## smh_smh       0.0102653154 0.0000000 0.000000000
## smm_vta       0.0000000000 0.0000000 0.000000000
## smm_cgc       0.0000000000 0.0000000 0.000000000
## smm_sa        0.0000000000 0.0000000 0.000000000
## smm_dla       0.0000000000 0.0000000 0.000000000
## smm_dt        0.0000000000 0.0000000 0.000000000
## smm_ca        0.0000000000 0.0000000 0.000000000
## smm_fo        0.0000000000 0.0000000 0.000000000
## smm_rspltp    0.0000000000 0.0000000 0.000000000
## smm_ad        0.0000000000 0.0000000 0.000000000
## smm_vs        0.0000000000 0.0000000 0.000000000
## smm_smh       0.0000000000 0.0000000 0.000000000
## smm_smm       0.0000000000 0.0000000 0.000000000
```

Combine into matrix and multiply by 100

```
imp <- 100 * cbind(LASSO = imp1, MCP = imp2, SCAD = imp3)
```

Extract brain connectivity importance indices

```
imp.brain <- imp[-c(1:8),]
```

Find active brain connectivity terms

```
actid <- (apply(imp.brain, 1, function(x) sqrt(sum(x^2))) > .Machine$double.eps)
```

Define colors for plot

```
cols <- c("#e41a1c", "#377eb8", "#4daf4a")
```

Plot importance indices

```
# save pdf
pdf(file = "fig4_imp.pdf", width = 8, height = 10)
par(mfrow = c(2,1), mar = c(4, 4.5, 4, 2) + 0.1)
barplot(t(imp[1:8,]), beside = TRUE, xlab = "", ylab = "Importance",
        main = "Demographic/Parental Variable Importance Indices", col = cols,
        ylim = c(0, 45), cex = 1.25, cex.axis = 1.25, cex.lab = 1.5, cex.main = 1.75)
box()
grid(nx = NA, ny = NULL)
legend("top", c("LASSO", "MCP", "SCAD"), fill = cols, bty = "n", cex = 1.25, horiz = TRUE)
temp <- barplot(t(imp.brain[actid,]), beside = TRUE, xlab = "", ylab = "Importance",
                main = "Brain Connectivity Variable Importance Indices", col = cols, xaxt = "n",
                ylim = c(0, 45), cex = 1.25, cex.axis = 1.25, cex.lab = 1.5, cex.main = 1.75)
box()
grid(nx = NA, ny = NULL)
text(x=temp[2,]-.25, y=-5.5, names(actid)[actid], xpd=TRUE, srt=45, cex = 1.25)
legend("top", c("LASSO", "MCP", "SCAD"), fill = cols, bty = "n", cex = 1.25, horiz = TRUE)
dev.off()
```

```
## quartz_off_screen 
##                 2
```

```
# print to html
par(mfrow = c(2,1), mar = c(4, 4.5, 4, 2) + 0.1)
barplot(t(imp[1:8,]), beside = TRUE, xlab = "", ylab = "Importance",
        main = "Demographic/Parental Variable Importance Indices", col = cols,
        ylim = c(0, 45), cex = 1.25, cex.axis = 1.25, cex.lab = 1.5, cex.main = 1.75)
box()
grid(nx = NA, ny = NULL)
legend("top", c("LASSO", "MCP", "SCAD"), fill = cols, bty = "n", cex = 1.25, horiz = TRUE)
temp <- barplot(t(imp.brain[actid,]), beside = TRUE, xlab = "", ylab = "Importance",
                main = "Brain Connectivity Variable Importance Indices", col = cols, xaxt = "n",
                ylim = c(0, 45), cex = 1.25, cex.axis = 1.25, cex.lab = 1.5, cex.main = 1.75)
box()
grid(nx = NA, ny = NULL)
text(x=temp[2,]-.25, y=-5.5, names(actid)[actid], xpd=TRUE, srt=45, cex = 1.25)
legend("top", c("LASSO", "MCP", "SCAD"), fill = cols, bty = "n", cex = 1.25, horiz = TRUE)
```

# Estimated Baseline CBCL Attenion Problems

Estimated baseline for LASSO model

```
exp(coef(mod1, s = "lambda.1se")[1])
```

```
## [1] 2.669299
```

Estimated baseline for MCP model

```
exp(coef(mod2, s = "lambda.1se")[1])
```

```
## [1] 1.452204
```

Estimated baseline for SCAD model

```
exp(coef(mod3, s = "lambda.1se")[1])
```

```
## [1] 1.675073
```

# Table 2: Estimated Demographic/Parental Effects

Build table 2

```
tab2 <- NULL
for(term in c("sex", "income", "alcohol", "drugs")){
  newdata <- abcd[1:nlevels(abcd[,term]),]
  newdata[,term] <- factor(levels(abcd[,term]), levels = levels(abcd[,term]),
                           ordered = is.ordered(abcd[,term]))
  fit1 <- predict(mod1, newdata = newdata, type = "terms")[,term]
  fit2 <- predict(mod2, newdata = newdata, type = "terms")[,term]
  fit3 <- predict(mod3, newdata = newdata, type = "terms")[,term]
  newtab <- as.data.frame(cbind(LASSO = exp(fit1), MCP = exp(fit2), SCAD = exp(fit3)))
  newtab <- cbind(term = term, levels = levels(abcd[,term]), round(newtab, 2))
  tab2 <- rbind(tab2, newtab)
}
rownames(tab2) <- 1:nrow(tab2)
write.csv(tab2, file = "tab2_imp.csv", row.names = FALSE)
```

Print table 2

```
knitr::kable(tab2)
```

| term | levels | LASSO | MCP | SCAD |
| --- | --- | --- | --- | --- |
| sex | male | 1.15 | 1.17 | 1.18 |
| sex | female | 0.87 | 0.85 | 0.85 |
| income | <50k | 1.10 | 1.13 | 1.16 |
| income | 50-100k | 0.99 | 0.99 | 0.99 |
| income | >100k | 0.92 | 0.89 | 0.87 |
| alcohol | none | 0.94 | 0.87 | 0.98 |
| alcohol | father | 0.96 | 0.93 | 0.99 |
| alcohol | mother | 1.00 | 1.03 | 1.00 |
| alcohol | both | 1.11 | 1.19 | 1.04 |
| drugs | none | 0.79 | 0.76 | 0.75 |
| drugs | father | 0.93 | 0.93 | 0.93 |
| drugs | mother | 1.10 | 1.12 | 1.12 |
| drugs | both | 1.24 | 1.27 | 1.29 |

# Table 3: Estimated Study Site Effect

Build table 3

```
# define new data
newdata <- abcd[1:22,]
newdata[,"site"] <- levels(abcd$site)

# obtain terms predictions
fit1 <- predict(mod1, newdata = newdata, type = "terms")[,"site"]
fit2 <- predict(mod2, newdata = newdata, type = "terms")[,"site"]
fit3 <- predict(mod3, newdata = newdata, type = "terms")[,"site"]

# combine into matrix
tab3 <- as.data.frame(cbind(LASSO = exp(fit1), MCP = exp(fit2), SCAD = exp(fit3)))
tab3 <- cbind(site = levels(abcd$site), round(tab3, 2))

# save result
rownames(tab3) <- 1:nrow(tab3)
write.csv(tab3, file = "tab3_site.csv", row.names = FALSE)
```

Print table 3

```
knitr::kable(tab3)
```

| site | LASSO | MCP | SCAD |
| --- | --- | --- | --- |
| site01 | 0.93 | 0.77 | 0.98 |
| site02 | 0.94 | 0.81 | 0.98 |
| site03 | 1.09 | 1.37 | 1.03 |
| site04 | 1.05 | 1.17 | 1.01 |
| site05 | 1.01 | 1.06 | 1.00 |
| site06 | 0.99 | 0.97 | 1.00 |
| site07 | 1.04 | 1.15 | 1.01 |
| site08 | 1.02 | 1.09 | 1.01 |
| site09 | 0.97 | 0.95 | 0.99 |
| site10 | 0.94 | 0.79 | 0.98 |
| site11 | 1.04 | 1.14 | 1.01 |
| site12 | 1.04 | 1.13 | 1.01 |
| site13 | 0.97 | 0.90 | 0.99 |
| site14 | 0.94 | 0.80 | 0.98 |
| site15 | 1.04 | 1.12 | 1.01 |
| site16 | 1.02 | 1.13 | 1.01 |
| site17 | 0.99 | 0.96 | 1.00 |
| site18 | 0.98 | 0.91 | 0.99 |
| site19 | 0.96 | 0.86 | 0.99 |
| site20 | 1.00 | 1.04 | 1.00 |
| site21 | 1.01 | 1.05 | 1.00 |
| site22 | 1.03 | 1.08 | 1.01 |

# Figure 5: Estimated DT-DLA Connectivity Effect

Define new data

```
newdata <- abcd[1:200,]
newdata[,"dt_dla"] <- seq(min(abcd$dt_dla), max(abcd$dt_dla), length.out = 200)
```

Obtain terms predictions

```
fit1 <- predict(mod1, newdata = newdata, type = "terms")[,"dt_dla"]
fit2 <- predict(mod2, newdata = newdata, type = "terms")[,"dt_dla"]
fit3 <- predict(mod3, newdata = newdata, type = "terms")[,"dt_dla"]
```

Plot DT-DLA effect on link and response scale

```
# save pdf
pdf(file = "fig5_dtdla.pdf", width = 8, height = 10)
par(mfrow = c(2,1), mar = c(4.5, 4.5, 4, 2) + 0.1)
plot(newdata$dt_dla, fit1, type = "l", lwd = 3, col = cols[1],
     cex = 1.25, cex.axis = 1.25, cex.lab = 1.5, cex.main = 1.75,
     ylim = extendrange(c(fit1,fit2,fit3)),
     xlab = "DT-DLA Functional Connectivity", ylab = "log(CBCL Attn Prob)",
     main = "Predictions on Link Scale")
lines(newdata$dt_dla, fit2, type = "l", lwd = 3, col = cols[2], lty = 2)
lines(newdata$dt_dla, fit3, type = "l", lwd = 3, col = cols[3], lty = 4)
grid()
legend("bottomright", c("LASSO", "MCP", "SCAD"), lty = c(1,2,4),
       cex = 1.25, lwd = 3, col = cols, bty = "n")
plot(newdata$dt_dla, exp(fit1),
     type = "l", lwd = 3, col = cols[1],
     cex = 1.25, cex.axis = 1.25, cex.lab = 1.5, cex.main = 1.75,
     ylim = extendrange(exp(c(fit1,fit2,fit3))),
     xlab = "DT-DLA Functional Connectivity", ylab = "CBCL Attn Prob",
     main = "Predictions on Response Scale")
lines(newdata$dt_dla, exp(fit2),
      type = "l", lwd = 3, col = cols[2], lty = 2)
lines(newdata$dt_dla, exp(fit3),
      type = "l", lwd = 3, col = cols[3], lty = 4)
grid()
legend("bottomright", c("LASSO", "MCP", "SCAD"), lty = c(1,2,4),
       cex = 1.25, lwd = 3, col = cols, bty = "n")
dev.off()
```

```
## quartz_off_screen 
##                 2
```

```
# print to html
par(mfrow = c(2,1), mar = c(4.5, 4.5, 4, 2) + 0.1)
plot(newdata$dt_dla, fit1, type = "l", lwd = 3, col = cols[1],
     cex = 1.25, cex.axis = 1.25, cex.lab = 1.5, cex.main = 1.75,
     ylim = extendrange(c(fit1,fit2,fit3)),
     xlab = "DT-DLA Functional Connectivity", ylab = "log(CBCL Attn Prob)",
     main = "Predictions on Link Scale")
lines(newdata$dt_dla, fit2, type = "l", lwd = 3, col = cols[2], lty = 2)
lines(newdata$dt_dla, fit3, type = "l", lwd = 3, col = cols[3], lty = 4)
grid()
legend("bottomright", c("LASSO", "MCP", "SCAD"), lty = c(1,2,4),
       cex = 1.25, lwd = 3, col = cols, bty = "n")
plot(newdata$dt_dla, exp(fit1),
     type = "l", lwd = 3, col = cols[1],
     cex = 1.25, cex.axis = 1.25, cex.lab = 1.5, cex.main = 1.75,
     ylim = extendrange(exp(c(fit1,fit2,fit3))),
     xlab = "DT-DLA Functional Connectivity", ylab = "CBCL Attn Prob",
     main = "Predictions on Response Scale")
lines(newdata$dt_dla, exp(fit2),
      type = "l", lwd = 3, col = cols[2], lty = 2)
lines(newdata$dt_dla, exp(fit3),
      type = "l", lwd = 3, col = cols[3], lty = 4)
grid()
legend("bottomright", c("LASSO", "MCP", "SCAD"), lty = c(1,2,4),
       cex = 1.25, lwd = 3, col = cols, bty = "n")
```
